# Association of Sociodemographic Factors With Overtriage, Undertriage, and Value of Care After Major Surgery

**DOI:** 10.1097/AS9.0000000000000429

**Published:** 2024-05-01

**Authors:** Tyler J. Loftus, Matthew M. Ruppert, Benjamin Shickel, Tezcan Ozrazgat-Baslanti, Jeremy A. Balch, Kenneth L. Abbott, Die Hu, Adnan Javed, Firas Madbak, Faheem Guirgis, David Skarupa, Philip A. Efron, Patrick J. Tighe, William R. Hogan, Parisa Rashidi, Gilbert R. Upchurch, Azra Bihorac

**Affiliations:** From the * Intelligent Critical Care Center, University of Florida, Gainesville, FL; † Department of Surgery, University of Florida Health, Gainesville, FL; ‡ Department of Medicine, University of Florida Health, Gainesville, FL; § Departments of Biomedical Engineering, Computer and Information Science and Engineering, and Electrical and Computer Engineering, University of Florida, Gainesville, FL; ∥ Departments of Emergency Medicine & Critical Care Medicine, University of Florida College of Medicine, Jacksonville, FL; ¶ Department of Surgery, University of Florida College of Medicine, Jacksonville, FL; # Department of Emergency Medicine, University of Florida College of Medicine, Jacksonville, FL; ** Departments of Anesthesiology, Orthopedics, and Information Systems/Operations Management, University of Florida Health, Gainesville, FL; †† Department of Health Outcomes & Biomedical Informatics, College of Medicine, University of Florida, Gainesville, FL.

**Keywords:** decision, ICU, phenotype, surgery, triage

## Abstract

**Objective:**

To determine whether certain patients are vulnerable to errant triage decisions immediately after major surgery and whether there are unique sociodemographic phenotypes within overtriaged and undertriaged cohorts.

**Background:**

In a fair system, overtriage of low-acuity patients to intensive care units (ICUs) and undertriage of high-acuity patients to general wards would affect all sociodemographic subgroups equally.

**Methods:**

This multicenter, longitudinal cohort study of hospital admissions immediately after major surgery compared hospital mortality and value of care (risk-adjusted mortality/total costs) across 4 cohorts: overtriage (N = 660), risk-matched overtriage controls admitted to general wards (N = 3077), undertriage (N = 2335), and risk-matched undertriage controls admitted to ICUs (N = 4774). K-means clustering identified sociodemographic phenotypes within overtriage and undertriage cohorts.

**Results:**

Compared with controls, overtriaged admissions had a predominance of male patients (56.2% vs 43.1%, *P* < 0.001) and commercial insurance (6.4% vs 2.5%, *P* < 0.001); undertriaged admissions had a predominance of Black patients (28.4% vs 24.4%, *P* < 0.001) and greater socioeconomic deprivation. Overtriage was associated with increased total direct costs [$16.2K ($11.4K–$23.5K) vs $14.1K ($9.1K–$20.7K), *P* < 0.001] and low value of care; undertriage was associated with increased hospital mortality (1.5% vs 0.7%, *P* = 0.002) and hospice care (2.2% vs 0.6%, *P* < 0.001) and low value of care. Unique sociodemographic phenotypes within both overtriage and undertriage cohorts had similar outcomes and value of care, suggesting that triage decisions, rather than patient characteristics, drive outcomes and value of care.

**Conclusions:**

Postoperative triage decisions should ensure equality across sociodemographic groups by anchoring triage decisions to objective patient acuity assessments, circumventing cognitive shortcuts and mitigating bias.

## Abstract 2

Mini-abstract:

In this multicenter, longitudinal cohort study, low-acuity patients overtriaged to intensive care units immediately after surgery had high proportions of male patients and commercial insurance; high-acuity patients undertriaged to general wards had a predominance of Black patients and greater socioeconomic deprivation. Postoperative triage decision-making should ensure sociodemographic equality by anchoring triage decisions to objective patient acuity assessments.

## INTRODUCTION

For the more than 300 million surgeries performed globally each year, surgeons must decide whether patients require admission to a resource-rich intensive care unit (ICU) or to a lower-acuity general hospital ward.^1^ When making triage decisions, doctors must accurately predict the risk for postoperative adverse events and the need for ICU resources, often relying exclusively on individual judgment under time constraints. Unsurprisingly, postoperative triage decisions occasionally err. Postoperative undertriage of high-acuity patients to hospital wards is associated with increased mortality and morbidity; overtriage of low-acuity patients to ICUs is associated with low value of care (outcomes/costs).^2–4^

In an equitable healthcare system, postoperative overtriage and undertriage would be driven exclusively by overestimation or underestimation of patients’ risk for adverse events and the need for ICU resources. Conversely, prior work suggests that sociodemographic factors may be associated with postoperative triage decisions.^2,3^ It remains unknown whether some groups of patients are especially vulnerable to triage decision errors and whether there are unique sociodemographic phenotypes within groups of overtriaged and undertriaged patients; this study seeks to address these knowledge gaps and serve as a scientific foundation for ensuring equality in future postoperative triage decision support systems.

This study uses a longitudinal, retrospective cohort of postoperative admissions at 3 university hospitals to test associations among sociodemographic factors, overtriage, undertriage, patient outcomes, and value of care. The aim of this study was to apply a clustering algorithm to overtriaged and undertriaged admissions to test the hypothesis that there are unique sociodemographic phenotypes within overtriaged and undertriaged populations.

## METHODS

### Study Design

This observational study uses a longitudinal, retrospective cohort of 10,846 postoperative admissions for patients age 18 years or greater at (1) the University of Florida Health Gainesville, a 1162-bed quaternary care center, (2) University of Florida Health Jacksonville, a 695-bed university hospital within the urban core of Jacksonville that is approximately 73 miles away from the Gainesville hospital, and (3) University of Florida Health North, a 92-bed university hospital in a community setting that is 11 miles away from the main Jacksonville campus. Institutional Review Board approval was obtained (IRB# 201802284). This study complies with strengthening the reporting of observational studies in epidemiology guidelines (Supplemental Table 1, see http://links.lww.com/AOSO/A343).

### Population and Data Source

The study population was derived by harmonizing datasets from 3 medical centers for which postoperative triage classifications (ie, overtriage, undertriage, and appropriate triage) have been previously developed and validated, as illustrated in Supplemental Figure 2, see http://links.lww.com/AOSO/A343, including a 6-year period ending September 2020. Briefly, a deep learning algorithm used input features from electronic health records to generate predictions of (1) hospital mortality and (2) prolonged (48 hours or greater) ICU stay using data available at the end of surgery.

### Model Features

The features, as described in Supplemental Methods 3, see http://links.lww.com/AOSO/A343, included patient demographics, hospital stations (eg, operating room, ICU, and ward), vital signs, ventilator settings, laboratory values, medications, blood product transfusions, diagnoses, operative procedures (classified as emergent, urgent, or elective rather than day of the week or daytime versus nighttime procedure) and procedural information, and indicators of illness severity [American Society of Anesthesiologists (ASA) Physical Status Classification scores, Charlson comorbidity index scores, and Sequential Organ Failure Assessment (SOFA) scores].^2,4^ Admissions with missing data were excluded unless otherwise noted in the results, as reported by the raw number and percentage of admissions with missing or unknown values.

Feature selection was performed by searching for pairs of correlated variables, calculating their mutual information scores, and discarding the variable with a lower score. Thus, selected variables had high information scores and low intervariable correlation. In validation cohorts, hospital mortality and prolonged ICU stay predictions had areas under the receiver operating characteristic curve of 0.92 [95% confidence interval (CI) = 0.91–0.93] and 0.92 (95% CI = 0.92–0.92), respectively.

### Primary Outcomes

These predictions were used to identify high-acuity patients (ie, deep learning predictions corresponding to the top quartile risk for either hospital mortality or prolonged ICU stay) who were undertriaged to general wards and to identify low-acuity patients (ie, deep learning predictions corresponding to below 50th percentile risk for both hospital mortality and prolonged ICU stay) who were overtriaged to ICUs, based on risk thresholds from published literature, as previously described.^2,4^

Aggregate results from the present study population have never been reported. The first surgery of each admission was considered in the main analysis. Deaths during the index surgery and admissions lasting less than 24 hours were excluded.

### Cohort Comparisons

Overtriaged ICU admissions were compared with controls that had similar risk profiles but were triaged to general wards; undertriaged ward admissions were compared with controls that had similar risk profiles but were triaged to ICUs. Risk-matched controls were identified by using a K-nearest neighbors matching algorithm, as previously described. The final dataset included 4 groups representing overtriaged admissions (N = 660), risk-matched controls for overtriaged admissions (N = 3077), undertriaged admissions (N = 2335), and risk-matched controls for undertriaged admissions (N = 4774).

As previously described, the intermediate care unit and ICU beds were considered together as a single ICU entity that provides low patient-to-nurse ratios, specialized critical care resources and personnel, and daily rounds by critical care attending.^4^ This approach also allows for accurate representation of total ICU bed occupancy, which has important implications for denied ICU referrals.^5^ Data regarding telemetry and continuous pulse oximetry among ward patients were inconsistently available at 2 of the hospitals and were therefore not analyzed. Previous sensitivity analyses exploring whether skin and soft tissue cases performed by Burn Surgery, Plastic and Reconstructive Surgery, and Otolaryngology affect overtriage classifications demonstrated that associations between triage classifications and patient outcomes were similar to those observed in the overall cohort.^4^ Therefore, these skin and soft tissue cases were included in the present analysis. In addition, CPT codes are input features in the prediction models that are used to generate triage classifications.

### Sample Size Determination

Sample size determination was performed using results from a triage classification external validation study, in which the incidence of hospital mortality or discharge to hospice was 4.2% among undertriaged admissions and 1.4% among risk-matched controls with an imbalanced sampling ratio of approximately 2.4 controls for each undertriaged admission, demonstrating that 459 undertriaged admissions would be required to obtain 80% power with alpha = 0.05.^6^ Mean value of care was 0.23 among overtriaged admissions and 1.71 among risk-matched controls with standard deviation 1.11 with an imbalanced sampling ratio of approximately 4.3 controls for each overtriaged admissions, such that 544 overtriaged admissions would be required to obtain 80% power with alpha = 0.05.^6^

### Clustering Algorithm

To understand whether there were unique sociodemographic phenotypes within groups of overtriaged and undertriaged patients, a clustering algorithm was applied to separate groups of overtriaged admissions and undertriaged admissions, using all available sociodemographic data (ie, age, sex, race, area deprivation index values, and payer). Clustering was performed via the open-source K-means clustering algorithm, as described in Supplemental Methods 3, see http://links.lww.com/AOSO/A343, a description of supplemental methods. K-means was chosen as the clustering approach because it offers unique advantages for exploratory classifications of patient states.^7^ Like other centroid-based clustering algorithms, K-means is sensitive to outliers; the source datasets for this study were subjected to outlier imputation, defining observations in the top and bottom 1% of each continuous variable’s distribution as outliers, as previously described.^2,3,8^ The optimal number of clusters was determined by calculating the within-cluster variance for a range of 1–9 clusters and identifying the inflection point at which a greater number of clusters (and attendant decrease in cluster sizes) would not substantially tighten the clusters (decrease the within-cluster sum of squares). Value of care, the primary outcome, was calculated as inverted observed-to-expected mortality ratios divided by median total costs and multiplied by a constant, as previously described.^2,4,9^ Other statistical analyses are described in Supplemental Methods 3, see http://links.lww.com/AOSO/A343.

## RESULTS

### Overtriaged and Undertriaged Admissions Had Unique Sociodemographic and Illness Severity Characteristics

The sociodemographic characteristics of the entire study population are listed in Table 1. The overtriage cohort had the greatest proportion of males (overtriage: 56.2%; overtriage controls: 43.1%, *P* < 0.001; undertriage: 50.8%, *P* = 0.02), younger age [overtriage: 52 (36–64); undertriage: 62 (52–73) years, *P* < 0.001], the greatest proportion with commercial insurance (overtriage: 6.4%; overtriage controls: 2.5%, *P* < 0.001; undertriage: 1.9%, *P* <0.001), and lesser proportions with Medicare (overtriage: 18.9%; undertriage: 37.3%, *P* < 0.001) and Medicare health maintenance organization (HMO) (overtriage: 12.1%; undertriage: 22.6%, *P* < 0.001). The undertriage cohort had the highest proportion of admissions in which the patient self-identified as Black or African American (undertriage: 28.4%; undertriage controls: 24.4%, *P* < 0.001; overtriage: 18.9%, *P* < 0.001) and the lowest proportion of White patients (undertriage: 65.1%; undertriage controls: 69.3%, *P* < 0.001; overtriage: 73.6%, *P* < 0.001). The undertriage cohort also had higher area deprivation index national rank values [undertriage: 72.0 (52.0–87.0); undertriage controls: 70.0 (50.0–87.0), *P* = 0.03; overtriage: 68.0 (46.0–83.2), *P* < 0.001] and the lowest proportions with Blue Cross Blue Shield (undertriage: 8.8%; undertriage controls: 13.3%, *P* < 0.001; overtriage: 17.6%, *P* < 0.001), self-pay (undertriage: 3.1%; undertriage controls: 4.3%, *P* = 0.02; overtriage: 6.4%, *P* < 0.001), and Workers’ compensation (undertriage: 0.4%; undertriage controls: 0.9%, *P* < 0.001; overtriage: 2.0%, *P* < 0.001).

**TABLE 1.** Sociodemographic Characteristics of Postoperative Admissions That Were Overtriaged to Intensive Care Units, Undertriaged to Hospital Wards, and Risk-Matched Controls

Sociodemographic Characteristics	Overtriage N = 660 No (%)	Overtriage Control N = 3077 No (%)	Undertriage N = 2335 No (%)	Undertriage Controls N = 4774 No (%)	*P* *	*P* †	*P* ‡
Female	289 (43.8)	1750 (56.9)	1148 (49.2)	2043 (42.8)	<0.001	<0.001	0.02
Male	371 (56.2)	1327 (43.1)	1187 (50.8)	2731 (57.2)	<0.001	<0.001	0.02
Age (years), median [IQR]	52 [36–64]	54 [37–67]	62 [52–73]	60 [46.2–70]	0.07	<0.001	<0.001
Race							
American Indian or Alaska Native	2 (0.3)	4 (0.1)	2 (0.1)	7 (0.1)	0.29	0.73	0.21
Asian	5 (0.8)	38 (1.2)	16 (0.7)	31 (0.6)	0.42	0.88	0.79
Black or African American	125 (18.9)	644 (20.9)	663 (28.4)	1165 (24.4)	0.27	<0.001	<0.001
Native Hawaiian or Other Pacific Islander	0 (0.0)	0 (0.0)	0 (0.0)	5 (0.1)	>0.99	0.18	>0.99
Other or multiracial	38 (5.8)	208 (6.8)	112 (4.8)	214 (4.5)	0.39	0.55	0.31
White	486 (73.6)	2155 (70.0)	1521 (65.1)	3310 (69.3)	0.07	<0.001	<0.001
Unknown	4 (0.6)	28 (0.9)	21 (0.9)	42 (0.9)	0.64	>0.99	0.63
Rural residence	167 (25.3)	818 (26.6)	575 (24.6)	965 (20.2)	0.77	<0.001	0.72
Area deprivation index							
State rank, median [IQR]	7.0 [5.0–9.0]	7.0 [5.0–9.0]	8.0 [5.0–9.0]	8.0 [5.0–9.0]	0.83	0.03	0.001
National rank, median [IQR]	68.0 [46.0–83.2]	68.0 [46.0–85.0]	72.0 [52.0–87.0]	70.0 [50.0–87.0]	0.97	0.03	<0.001
Social vulnerability index, median [IQR]	0.57 [0.41–0.69]	0.59 [0.41–0.68]	0.60 [0.43–0.70]	0.60 [0.43–0.73]	0.82	0.59	0.007
Payer							
Blue Cross Blue Shield	116 (17.6)	597 (19.4)	206 (8.8)	634 (13.3)	0.30	<0.001	<0.001
CMS	1 (0.2)	2 (0.1)	4 (0.2)	5 (0.1)	0.44	0.49	>0.99
Commercial	42 (6.4)	78 (2.5)	44 (1.9)	226 (4.7)	<0.001	<0.001	<0.001
Federal non-CMS	28 (4.2)	91 (3.0)	64 (2.7)	169 (3.5)	0.11	0.08	0.06
Managed care	67 (10.2)	283 (9.2)	107 (4.6)	285 (6.0)	0.46	0.02	<0.001
Medicaid	21 (3.2)	125 (4.1)	92 (3.9)	198 (4.1)	0.32	0.70	0.42
Medicaid HMO	78 (11.8)	398 (12.9)	246 (10.5)	476 (10.0)	0.48	0.48	0.36
Medicare	125 (18.9)	669 (21.7)	870 (37.3)	1,343 (28.1)	0.12	<0.001	<0.001
Medicare HMO	80 (12.1)	441 (14.3)	527 (22.6)	822 (17.2)	0.15	<0.001	<0.001
Self-pay	42 (6.4)	207 (6.7)	72 (3.1)	207 (4.3)	0.80	0.01	<0.001
Workers’ compensation	13 (2.0)	37 (1.2)	10 (0.4)	45 (0.9)	0.13	0.02	<0.001
Other or unknown	47 (7.1)	149 (4.8)	93 (4.0)	364 (7.6)	0.02	<0.001	0.002

*P* values correspond to significance tests comparing cohorts by each variable in the “Sociodemographic characteristics” column.

* Overtriage versus overtriage controls, which were ward admissions with risk profiles similar to those of overtriaged intensive care unit admissions.

† Undertriage versus undertriage controls, which were ICU admissions with risk profiles similar to those of undertriaged ward admissions.

‡ Overtriage versus undertriage.

CMS, Centers for Medicare & Medicaid Services; HMO, health maintenance organization; IQR, interquartile range.

Illness severity indicators of the entire study population are listed in Supplemental Table 4, see http://links.lww.com/AOSO/A343. Compared with overtriage controls, the overtriage cohort had higher SOFA scores on admission and immediately before surgery, but lower ASA scores, and the 2 groups had similar Charlson comorbidity index scores and similar incidence of emergent admission priority, emergent surgery, and both preoperative and intraoperative red cell transfusion. Compared with undertriage controls, the undertriage cohort had lower SOFA scores on admission and immediately before surgery, but higher ASA scores and Charlson comorbidity index scores, and had higher incidence of emergent admission priority, emergent surgery, and preoperative red cell transfusion. Primary surgical services of the entire study population are listed in Supplemental Table 5, see http://links.lww.com/AOSO/A343. There were disproportionate contributions to the overtriage cohort from Burn Surgery, Neurosurgery, Pancreas and Biliary Surgery, and Otolaryngology. There were disproportionate contributions to the undertriage cohort from Podiatry, Transplant Surgery, and Vascular Surgery.

### Overtriaged and Undertriaged Admissions Had Lower Value of Care Compared With Risk-Matched Controls

**FIGURE 1.**
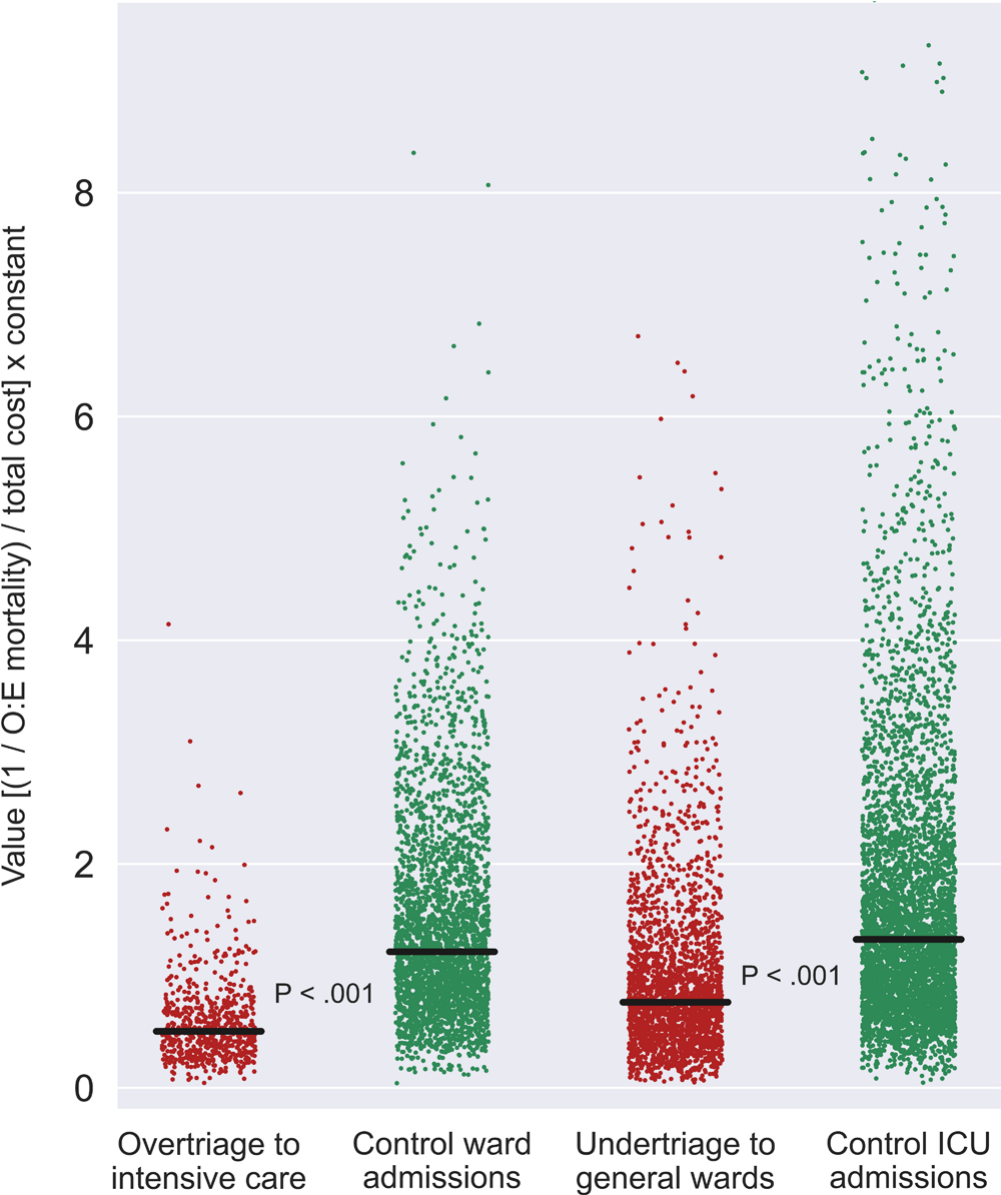
Both overtriage and undertriage were associated with low value of care relative to risk-matched controls. Each point represents a single admission. Black lines represent medians. *P* values compare nonparametric distributions between cohorts. To calculate value of care for each admission, inverted O:E mortality ratios for cohorts were divided by total cost for each admission and multiplied by a constant to set value of care for overtriage versus controls and undertriage versus controls to 1. Both hospital mortality and discharge to inpatient or outpatient hospice were considered observed mortalities. ICU indicates intensive care unit; O:E mortality, observed-to-expected hospital mortality ratios.

Outcomes of the entire study population are listed in Table 2. Compared with overtriage controls, overtriaged admissions had a greater incidence of prolonged ICU stay (22.4% vs 1.1%, *P* < 0.001), the longer length of stay in the ICU [1.0 (0.4–1.9) vs 0.0 (0.0–0.0) days, *P* < 0.001) and in the hospital [1.9 (1.1–3.0) vs 1.7 (1.0–2.9) days, *P* = 0.05), greater incidence of hospital mortality (0.3% vs 0.0%, *P* = 0.04) but the similar incidence of discharge to hospice (0.0% vs 0.2%, *P* = 0.62), higher total direct costs for the hospital admission [$16.2K ($11.4K–$23.5K) vs $14.1K ($9.1K–$20.7K), *P* < 0.001], and lower value of care [0.5 (0.3–0.7) vs 1.2 (0.8–1.9), *P* < 0.001, as illustrated in Fig. 1]. Compared with undertriage controls, the undertriage cohort had a greater incidence of red cell transfusion after surgery (16.8% vs 11.6%, *P* < 0.001) and during admission (21.7% vs. 14.2%, *P* < 0.001), greater incidence of persistent (lasting 72 hours or greater) acute kidney injury both with recovery before discharge (6.8% vs 3.9%, *P* < 0.001) and without recovery before discharge (6.2% vs 4.3%, *P* = 0.001), shorter ICU length of stay [0.0 (0.0–0.0) vs 2.0 (0.9–4.5) days, *P* < 0.001] but longer hospital length of stay [4.3 (2.5–7.6) vs 3.4 (1.8–5.8) days, *P* < 0.001], greater incidence of hospital mortality (1.5% vs 0.7%, *P* = 0.002) and discharge to hospice (2.2% vs 0.6%, *P* < 0.001), lower total costs [$24.7K ($15.9K–$39.4K) vs $27.9K ($17.7K–$43.9K), *P* < 0.001], and lower value of care [0.8 (0.5–1.2) vs 1.3 (0.8–2.1), *P* < 0.001, as illustrated in Fig. 1].

**TABLE 2.** Outcomes of Postoperative Admissions That Were Overtriaged to Intensive Care Units, Undertriaged to Hospital Wards, and Risk-Matched Controls

Outcomes	Overtriage N = 660 No (%)	Overtriage Controls N = 3077 No (%)	Undertriage N = 2335 No (%)	Undertriage Controls N = 4774 No (%)	*P* *	*P* †	*P* ‡
Second surgery during admission	56 (8.5)	134 (4.4)	475 (20.3)	697 (14.6)	<0.001	<0.001	<0.001
Hours between surgeries, median [IQR]	66 [43–97]	72 [38–123]	90 [48–145]	73 [39–144]	0.49	0.02	0.02
Emergent second surgery	8 (1.2)	26 (0.8)	117 (5.0)	163 (3.4)	0.41	0.002	<0.001
Had postoperative red cell transfusion	30 (4.5)	98 (3.2)	392 (16.8)	556 (11.6)	0.11	<0.001	<0.001
Red cell transfusion during admission	30 (4.5)	122 (4.0)	507 (21.7)	678 (14.2)	0.56	<0.001	<0.001
AKI with rapid reversal	45 (6.8)	138 (4.5)	275 (11.8)	493 (10.3)	0.02	0.08	<0.001
Persistent AKI with renal recovery	5 (0.8)	29 (0.9)	159 (6.8)	185 (3.9)	0.838	<0.001	<0.001
Persistent AKI without renal recovery	27 (4.1)	83 (2.7)	144 (6.2)	204 (4.3)	0.07	0.001	0.06
ICU admission for ≥48 hours	148 (22.4)	34 (1.1)	222 (9.5)	2398 (50.2)	<0.001	<0.001	<0.001
Mechanical ventilation for ≥48 hours	3 (0.5)	18 (0.6)	51 (2.2)	93 (1.9)	>0.99	0.56	0.002
ICU length of stay (days), median [IQR]	1.0 [0.4–1.9]	0.0 [0.0–0.0]	0.0 [0.0–0.0]	2.0 [0.9–4.5]	<0.001	<0.001	<0.001
Hospital length of stay (days), median [IQR]	1.9 [1.1–3.0]	1.7 [1.0–2.9]	4.3 [2.5–7.6]	3.4 [1.8–5.8]	0.05	<0.001	<0.001
Hospital mortality	2 (0.3)	0 (0.0)	36 (1.5)	33 (0.7)	0.04	0.002	0.01
Discharge to hospice	0 (0.0)	5 (0.2)	51 (2.2)	31 (0.6)	0.62	<0.001	<0.001
Professional service charges, $K, median [IQR]	13.4 [9.5–20.0]	11.8 [7.7–16.9]	15.9 [10.2–25.9]	20.3 [12.3–34.4]	<0.001	<0.001	<0.001
Hospital admission charges, $K, median [IQR]	70.3 [49.9–103.0]	63.7 [43.8–88.1]	104.8 [70.5–161.7]	118.6 [80.5–171.6]	<0.001	<0.001	<0.001
Hospital admission costs, $K, median [IQR]	16.2 [11.4–23.5]	14.1 [9.1–20.7]	24.7 [15.9–39.4]	27.9 [17.7–43.9]	<0.001	<0.001	<0.001
Value of care, median [IQR]	0.5 [0.3–0.7]	1.2 [0.8–1.9]	0.8 [0.5–1.2]	1.3 [0.8–2.1]	<0.001	<0.001	<0.001

*P* values were adjusted for multiple comparisons using the Benjamini-Hochberg procedure. *P* values correspond to significance tests comparing cohorts by each variable in the “Outcomes” column.

* Overtriage versus overtriage controls, which were ward admissions with risk profiles similar to those of overtriaged intensive care unit admissions.

† Undertriage versus undertriage controls, which were ICU admissions with risk profiles similar to those of undertriaged ward admissions.

‡ Overtriage versus undertriage.

AKI indicates acute kidney injury; ICU, intensive care unit; IQR, interquartile range.

### Overtriage and Undertriage Phenotyping

For both overtriage and undertriage phenotyping, the optimal number of clusters was 3, as illustrated in Supplemental Figure 6, see http://links.lww.com/AOSO/A343. T-distributed stochastic neighbor embedding plots of overtriage and undertriage phenotypes are illustrated in Supplemental Figure 7, see http://links.lww.com/AOSO/A343. The overtriage phenotypes were arbitrarily named alpha (N = 233), beta (N = 218), and gamma (N = 209); the undertriage phenotypes were named delta (N = 992), epsilon (N = 820), and zeta (N = 523).

### Overtriage Phenotypes Had Significant Sociodemographic and Illness Severity Differences But Similar Outcomes

Sociodemographic characteristics of overtriage phenotypes are listed in Table 3. The alpha phenotype had the greatest age [alpha: 62 (56–70) years; beta: 55 (41–67) years, *P* < 0.001; gamma: 33 (27–41) years, *P* < 0.001] and the greatest proportion with Medicare HMO (alpha: 22.3%; beta: 10.1%, *P* < 0.001; gamma: 2.9%, *P* < 0.001). The beta phenotype had the lowest incidence of rural residence (beta: 17.0%; alpha: 31.3%; gamma: 27.3%, *P* < 0.001), the lowest area deprivation indices for both state ranks [beta: 4.0 (3.0–5.0); alpha: 8.0 (7.0–9.0), *P* < 0.001; gamma: 8.0 (8.0–10.0), *P* < 0.001] and national ranks [beta: 39.0 (30.2–46.0); alpha: 79.0 (67.0–89.0); gamma: 79.0 (70.0–90.0), *P* < 0.001], the lowest social vulnerability indices [beta: 0.41 (0.33–0.55); alpha: 0.62 (0.49–0.74); gamma: 0.62 (0.53–0.77), *P* < 0.001], and the highest proportion with Blue Cross Blue Shield (beta: 25.2%; alpha: 11.6%, *P* < 0.001; gamma: 16.3%, *P* = 0.02). The gamma phenotype had the lowest age, the highest proportion of Black patients (gamma: 34.0%; alpha: 14.6%, *P* < 0.001; beta: 9.2%, *P* < 0.001), the lowest proportion of White patients (gamma: 57.4%; alpha: 78.1%, *P* < 0.001; beta: 84.4%, *P* < 0.001), the highest proportion with Medicaid HMO (gamma: 19.1%; alpha: 10.7%, *P* = 0.02; beta: 6.0%, *P* < 0.001) and the lowest proportions with Medicare (gamma: 5.7%; alpha: 27.5%, *P* < 0.001; beta: 22.5%, *P* < 0.001) and Medicare HMO (gamma: 2.9%; alpha: 22.3%, *P* < 0.001; beta: 10.1%, *P* = 0.003).

**TABLE 3.** Sociodemographic Characteristics of Overtriage Phenotypes

Sociodemographic Characteristics	Alpha Phenotype N = 233 No (%)	Beta Phenotype N = 218 No (%)	Gamma Phenotype N = 209 No (%)	*P* *	*P* †	*P* ‡
Female	114 (48.9)	81 (37.2)	94 (45.0)	0.01	0.45	0.12
Male	119 (51.1)	137 (62.8)	115 (55.0)	0.01	0.45	0.12
Age (years), median [IQR]	62 [56–70]	55 [41–67]	33 [27–41]	<0.001	<0.001	<0.001
Race						
American Indian or Alaska Native	0 (0.0)	2 (0.9)	0 (0.0)	0.23	>0.99	0.50
Asian	2 (0.9)	3 (1.4)	0 (0.0)	0.68	0.50	0.25
Black or African American	34 (14.6)	20 (9.2)	71 (34.0)	0.08	<0.001	<0.001
Native Hawaiian or Other Pacific Islander	0 (0.0)	0 (0.0)	0 (0.0)	>0.99	>0.99	>0.99
Other or multiracial	13 (5.6)	8 (3.7)	17 (8.1)	0.38	0.35	0.06
White	182 (78.1)	184 (84.4)	120 (57.4)	0.09	<0.001	<0.001
Unknown	2 (0.9)	1 (0.5)	1 (0.5)	>0.99	>0.99	>0.99
Rural residence	73 (31.3)	37 (17.0)	57 (27.3)	<0.001	0.40	0.009
Area deprivation index						
State rank, median [IQR]	8.0 [7.0–9.0]	4.0 [3.0–5.0]	8.0 [8.0–10.0]	<0.001	0.38	<0.001
National rank, median [IQR]	79.0 [67.0–89.0]	39.0 [30.2–46.0]	79.0 [70.0–90.0]	<0.001	0.59	<0.001
Social vulnerability index, median [IQR]	0.62 [0.49–0.74]	0.41 [0.33–0.55]	0.62 [0.53–0.77]	<0.001	0.39	<0.001
Payer						
Blue Cross Blue Shield	27 (11.6)	55 (25.2)	34 (16.3)	<0.001	0.17	0.02
CMS	0 (0.0)	0 (0.0)	1 (0.5)	>0.99	0.47	0.49
Commercial	6 (2.6)	14 (6.4)	22 (10.5)	0.07	<0.001	0.16
Federal non-CMS	7 (3.0)	13 (6.0)	8 (3.8)	0.17	0.79	0.37
Managed care	18 (7.7)	25 (11.5)	24 (11.5)	0.20	0.20	>0.99
Medicaid	5 (2.1)	5 (2.3)	11 (5.3)	>0.99	0.12	0.13
Medicaid HMO	25 (10.7)	13 (6.0)	40 (19.1)	0.09	0.02	<0.001
Medicare	64 (27.5)	49 (22.5)	12 (5.7)	0.23	<0.001	<0.001
Medicare HMO	52 (22.3)	22 (10.1)	6 (2.9)	<0.001	<0.001	0.003
Self-pay	14 (6.0)	8 (3.7)	20 (9.6)	0.28	0.21	0.02
Workers’ compensation	3 (1.3)	4 (1.8)	6 (2.9)	0.72	0.32	0.54
Other or unknown	12 (5.2)	10 (4.6)	25 (12.0)	0.83	0.02	0.007

*P* values correspond to significance tests comparing cohorts by each variable in the “Sociodemographic characteristics” column.

* Alpha phenotype versus beta phenotype.

† Alpha phenotype versus gamma phenotype.

‡ Beta phenotype versus gamma phenotype.

CMS indicates Centers for Medicare & Medicaid Services; HMO, health maintenance organization; IQR, interquartile range.

Illness severity indicators of overtriage phenotypes are listed in Supplemental Table 8, see http://links.lww.com/AOSO/A343. The alpha phenotype had the highest ASA scores and Charlson comorbidity index scores. The gamma phenotype had the highest proportions with emergent admission priority and emergent surgery. The incidences of preoperative and intraoperative red cell transfusions were similar across all 3 phenotypes. Primary surgical services of overtriage phenotypes are listed in Supplemental Table 9, see http://links.lww.com/AOSO/A343. There were disproportionate contributions to the alpha phenotype from Vascular Surgery, disproportionate contributions to the beta phenotype from Neurosurgery, and a disproportionate contribution to the gamma phenotype from Trauma and Acute Care Surgery.

Patient outcomes of overtriage phenotypes are listed in Supplemental Table 10, see http://links.lww.com/AOSO/A343. The gamma phenotype had shorter ICU length of stay compared with the alpha phenotype [0.9 (0.0–1.8) vs 1.2 (0.8–2.0) days, *P* < 0.001] and had the lowest professional service charges [gamma: $11.3K ($8.2K–$16.8K); alpha: $13.9K ($10.6K–$20.1K), *P* = 0.004; beta: $14.8K ($10.3K–$21.7K), *P* = 0.002]. The 3 phenotypes had similar total charges and total costs and had similar incidences of all measured complications. The value of care was 0.5 in all 3 phenotypes (all *P* ≥ 0.10).

### Undertriage Phenotypes Had Significant Sociodemographic and Illness Severity Differences But Similar Outcomes

Sociodemographic characteristics of undertriage phenotypes are listed in Table 4. The delta phenotype had the greatest age [delta: 68 (61–76) years; epsilon: 65 (55–75) years, *P* < 0.001; zeta: 44 (32–51), *P* < 0.001], the highest incidence of rural residence (delta: 32.4%; epsilon: 18.3%; zeta: 19.9%, *P* < 0.001), the lowest proportions of Blue Cross Blue Shield (delta: 3.6%; epsilon: 12.2%, *P* = 0.003; zeta: 13.4%, *P* < 0.001) and managed care (delta: 2.6%; epsilon: 5.4%, *P* = 0.003; zeta: 7.1%, *P* < 0.001), and the highest proportion of Medicare HMO (delta: 31.7%; epsilon: 22.3%, *P* < 0.001; zeta: 5.7%, *P* < 0.001); 75.1% of the delta phenotype had Medicare or Medicare HMO. The epsilon phenotype had median age 65 and the lowest proportion of Black patients (epsilon: 11.7%; delta: 33.8%, *P* < 0.001; zeta: 44.4%, *P* < 0.001), the lowest social vulnerability indices [epsilon: 0.46 (0.36–0.60); delta: 0.64 (0.54–0.80); zeta: 0.63 (0.54–0.83), *P* < 0.001], the highest proportion of White patients (epsilon: 81.5%; delta: 61.0%, *P* < 0.001; zeta: 47.4%, *P* < 0.001), the lowest area deprivation indices for both state ranks [epsilon: 4.0 (3.0–6.0); delta: 9.0 (8.0–10.0), *P* < 0.001; zeta: 9.0 (8.0–10.0), *P* < 0.001] and national ranks [epsilon: 44.0 (35.0–53.0); delta: 84.0 (74.0–93.0), *P* < 0.001; zeta: 84.0 (72.0–93.0), *P* < 0.001] and the lowest proportion with commercial insurance (epsilon: 0.9%; delta: 2.0%, *P* = 0.05; zeta: 3.3%, *P* = 0.002); 75.7% of the epsilon phenotype had Blue Cross Blue Shield, Medicare, or Medicare HMO. The zeta phenotype had the lowest age, the highest proportion of Black patients, the lowest proportion of White patients, and the highest proportions with Medicaid (zeta: 6.9%; delta: 3.1%, *P* = 0.001; epsilon: 3.0%, *P* = 0.002), Medicaid HMO (zeta: 25.4%; delta: 7.0%, *P* < 0.001; epsilon: 5.4%, *P* < 0.001), and self-pay (zeta: 5.5%; delta: 2.4%, *P* = 0.003; epsilon: 2.3%, *P* = 0.002), and the lowest proportions with Medicare (zeta: 19.3%; delta: 43.4%, *P* < 0.001; epsilon: 41.2%, *P* < 0.001) and Medicare HMO (zeta: 5.7%; delta: 31.7%, *P* < 0.001; epsilon: 22.3%, *P* < 0.001).

**TABLE 4.** Sociodemographic Characteristics Among Undertriage Phenotypes

Sociodemographic Characteristics	Delta Phenotype N = 992 No (%)	Epsilon Phenotype N = 820 No (%)	Zeta Phenotype N = 523 No (%)	*P* *	*P* †	*P* ‡
Female	495 (49.9)	388 (47.3)	265 (50.7)	0.28	0.79	0.24
Male	497 (50.1)	432 (52.7)	258 (49.3)	0.28	0.79	0.24
Age (years), median [IQR]	68 [61–76]	65 [55–75]	44 [32–51]	<0.001	<0.001	<0.001
Race						
American Indian or Alaska Native	1 (0.1)	1 (0.1)	0 (0.0)	>0.99	>0.99	>0.99
Asian	7 (0.7)	3 (0.4)	6 (1.1)	0.53	0.39	0.10
Black or African American	335 (33.8)	96 (11.7)	232 (44.4)	<0.001	<0.001	<0.001
Native Hawaiian or Other Pacific Islander	0 (0.0)	0 (0.0)	0 (0.0)	>0.99	>0.99	>0.99
Other or Multiracial	37 (3.7)	42 (5.1)	33 (6.3)	0.17	0.03	0.39
White	605 (61.0)	668 (81.5)	248 (47.4)	<0.001	<0.001	<0.001
Unknown	7 (0.7)	10 (1.2)	4 (0.8)	0.33	>0.99	0.58
Rural residence	321 (32.4)	150 (18.3)	104 (19.9)	<0.001	<0.001	0.39
Area deprivation index						
State rank, median [IQR]	9.0 [8.0–10.0]	4.0 [3.0–6.0]	9.0 [8.0–10.0]	<0.001	0.42	<0.001
National rank, median [IQR]	84.0 [74.0–93.0]	44.0 [35.0–53.0]	84.0 [72.0–93.0]	<0.001	0.41	<0.001
Social vulnerability index, median [IQR]	0.64 [0.54–0.80]	0.46 [0.36–0.60]	0.63 [0.54–0.83]	<0.001	0.78	<0.001
Payer						
Blue Cross Blue Shield	36 (3.6)	100 (12.2)	70 (13.4)	<0.001	<0.001	0.56
CMS	1 (0.1)	0 (0.0)	3 (0.6)	>0.99	0.12	0.06
Commercial	20 (2.0)	7 (0.9)	17 (3.3)	0.05	0.16	0.002
Federal non-CMS	24 (2.4)	27 (3.3)	13 (2.5)	0.32	>0.99	0.51
Managed care	26 (2.6)	44 (5.4)	37 (7.1)	0.003	<0.001	0.20
Medicaid	31 (3.1)	25 (3.0)	36 (6.9)	>0.99	0.001	0.002
Medicaid HMO	69 (7.0)	44 (5.4)	133 (25.4)	0.17	<0.001	<0.001
Medicare	431 (43.4)	338 (41.2)	101 (19.3)	0.36	<0.001	<0.001
Medicare HMO	314 (31.7)	183 (22.3)	30 (5.7)	<0.001	<0.001	<0.001
Self-pay	24 (2.4)	19 (2.3)	29 (5.5)	>0.99	0.003	0.002
Workers’ compensation	1 (0.1)	8 (1.0)	1 (0.2)	0.01	>0.99	0.17
Other or unknown	15 (1.5)	25 (3.0)	53 (10.1)	0.04	<0.001	<0.001

*P* values correspond to significance tests comparing cohorts by each variable in the “Sociodemographic characteristics” column.

* Delta phenotype versus epsilon phenotype.

† Delta phenotype versus zeta phenotype.

‡ Epsilon phenotype versus zeta phenotype.

CMS indicates Centers for Medicare & Medicaid Services; HMO, health maintenance organization; IQR, interquartile range.

Illness severity indicators of undertriage phenotypes are listed in Supplemental Table 11, see http://links.lww.com/AOSO/A343. The delta phenotype had the highest ASA scores and Charlson comorbidity index scores. The epsilon phenotype had the greatest proportion of elective admission priority. The zeta phenotype had the highest admission and preoperative SOFA scores and the greatest incidence of emergent surgery. The incidences of preoperative and intraoperative red cell transfusions were similar across all 3 phenotypes. Primary surgical services of undertriage phenotypes are listed in Supplemental Table 12, see http://links.lww.com/AOSO/A343. There was a disproportionate contribution to the delta phenotype from Orthopedic Surgery and disproportionate contributions to the zeta phenotype from Gynecologic Surgery, Pediatric Surgery, and Podiatry.

Patient outcomes of undertriage phenotypes are listed in Supplemental Table 13, see http://links.lww.com/AOSO/A343. The delta phenotype had a longer ICU length of stay compared with the epsilon phenotype [4.5 (2.9–7.8) vs 4.0 (2.4–6.9) days, *P* = 0.007]. The 3 phenotypes had similar costs, charges, and incidence of all measured complications. The value of care ranged from 0.7 to 0.8 across all 3 phenotypes (all *P* ≥ 0.93).

## DISCUSSION

Consistent with prior work, postoperative overtriage was associated with increased costs, no improvements in any patient outcomes, higher incidence of acute kidney injury, and low value of care, while postoperative undertriage was associated with increased mortality and morbidity and low value of care. Unlike prior work, in the present study, overtriage was associated with slightly increased mortality, suggesting that overtriaged patients had risk factors for mortality that were not captured by EHR data but were apparent to astute clinicians who knew something the model couldn’t know (eg, an intraoperative complication that was not manifest by a change in intraoperative vital signs, or a concerning physical exam finding). This observation supports the contention that human reasoning and tuition must remain paramount in clinical decision-making, even if decisions are augmented by prediction models. Although it is difficult to establish causal factors for the observed increased incidence of acute kidney injury and mortality among overtriaged patients, 2 explanations seem plausible. First, approximately 49% of all ICU patients receive diuretic therapy at some point during their ICU admission, which has the potential to push some toward prerenal acute kidney injury, especially those with total body water overload but unrecognized intravascular hypovolemia.^10^ Second, patients who were classified as overtriaged may have had risk factors for acute kidney injury or mortality that were underappreciated or not represented by the model input features but were known to the clinicians who decided upon ICU admission. The present study builds on prior work by describing the unique sociodemographic characteristics of overtriaged and undertriaged patients, which indicate inequities in postoperative triage decisions. This study also identifies unique sociodemographic patterns within overtriaged and undertriaged cohorts in clusters that were consistent with clinical intuition, adhering to the notion from Preud’homme et al^11^ that “despite the immense progress enabled by artificial intelligence in recent years, human experience and intuition remain the best judge in cluster analysis.” Interestingly, the set of 3 overtriage phenotypes and the set of 3 undertriage phenotypes each contained one larger group with advanced age, high proportions with Medicare and Medicare HMO, and greater burden of comorbid disease, one middle-sized group with socioeconomic advantage, and one smaller group with high proportions of Black patients, high proportions with Medicaid and Medicaid HMO, and high incidence of emergent surgery. Despite these significant differences in sociodemographic and illness severity indicators, patient outcomes and value of care were similar across phenotypes, suggesting that triage decisions, rather than patient characteristics, have the strongest associations with observed differences in mortality, morbidity, and value of care after major surgery.

The observed postoperative triage decision-making disparities may have been influenced by heuristics (cognitive shortcuts) or implicit bias. Surgeons making frequent, high-stakes decisions with incomplete information are vulnerable to decision fatigue, manifest as procrastination, less persistence when facing adversity, and lower quality and quantity of mathematic calculations.^12–15^ These impairments are exacerbated by sleep deprivation, which is common among surgeons.^16,17^ Under time constraints and uncertainty, decisions are often influenced by heuristics that can lead to decision-making errors, especially for less experienced clinicians.^18–21^ Dependence upon heuristics may also exacerbate the effects of implicit or subconscious bias, which could explain the observed sociodemographic disparities in postoperative triage patterns.^22^ While clinicians exhibit volatile performance in estimating risk for complications and in making postoperative triage decisions, machine learning models demonstrate relatively stable and occasionally superior performance, suggesting opportunities to augment postoperative triage decisions with objective, machine learning-enabled patient acuity assessments.^3,23^

The authors are unaware of prior reports describing associations between sociodemographic factors, postoperative triage, and value of care, but prior work has used clustering analyses to understand disease pathophysiology and predict treatment response. Secondary analyses of acute respiratory distress syndrome trials have consistently identified hyper-inflammatory and hypo-inflammatory phenotypes that have different responses to targeted treatments.^24–27^ These acute respiratory distress syndrome phenotypes were identified by latent class analysis, which is a probabilistic, distribution-based clustering method, but similar work has also been performed with centroid-based clustering, as used in the present study. Seymour et al^28^ applied consensus K-means clustering to 29 clinical and laboratory variables among sepsis patients and identified 4 phenotypes with unique pathophysiologic biomarker signatures and outcomes. In a series of simulations varying the proportions of each phenotype, there were significant differences in treatment benefits and harms, especially for early goal-directed therapy and activated protein C.^29–31^ These prior investigations support the validity of clustering analyses to identify and understand potentially informative patient phenotypes within larger, heterogenous patient cohorts. More recently, Thongprayoon et al^32^ applied consensus K-means clustering to understand phenotypes of Black kidney transplant patients in the national United Network for Organ Sharing/Organ Procurement and Transplantation Network database and identified 4 phenotypes that had unique clinical characteristics and risk for allograft rejection, suggesting that different phenotypes may have unique responses to different clinical care strategies.

### Limitations

This study was limited by its retrospective design. The selection bias inherent to the retrospective investigation was mitigated by including all consecutive admissions meeting relatively broad inclusion criteria. In addition, the sociodemographic data presented herein is limited to that which is available in electronic health record data and therefore does not represent other sociodemographic factors that have been associated with healthcare delivery disparities, such as gender and sexual orientation.^33,34^ It is possible to use International Classification of Diseases codes to identify individuals who identify as noncisgender, but this method has reported a sensitivity of 4% using electronic health record data.^35^ Institutional quality checks on electronic health record data representing ethnicity suggested that the data are unreliable, and were therefore not reported. Sociodemographic factors were included as features in the machine learning models that generated patient acuity assessments, raising the possibility that observed associations between sociodemographic factors and patient outcomes represent a tautology. Finally, all clustering methods share the weakness that they can potentially find clusters even when natural clusters do not exist, underscoring the importance of applying human intuition and clinical expertise to clustering results. The human intuition and clinical experience applied in this study are subjective and represent a potential source of error.

## CONCLUSIONS

Overtriaged admissions (ie, low-acuity patients who were admitted to ICUs immediately after surgery and received low-value care) had a predominance of male patients with commercial insurance; undertriaged admissions (ie, high-acuity patients who were admitted to general wards immediately after surgery and had increased risk for mortality and morbidity) had disproportionately high numbers of female and Black patients, more socioeconomic deprivation, and approximately double the proportion with Medicare. Triage decisions, rather than patient characteristics, had the strongest associations with observed differences in mortality, morbidity, and value of care. These findings suggest that postoperative triage decision-making should ensure equality across sociodemographic groups by anchoring triage decisions to objective patient acuity assessments that depend on representations of pathophysiology rather than identity, circumventing cognitive shortcuts and attendant implicit bias. Real-time, machine learning-enabled patient acuity assessments that are integrated with existing digital workflows have the potential to achieve this important goal.

## Supplementary Material


