# Supplementary material for: Association of Sociodemographic Factors With Overtriage, Undertriage, and Value of Care After Major Surgery
Source: Ann Surg Open. 2024 May 1;5(2):e429. doi: 10.1097/AS9.0000000000000429 (PMC11191932; doi:10.1097/AS9.0000000000000429)
Supplement: Supplementary file 1 [file as9-5-e429-s001.pdf]

Supplemental Digital Content 1. STROBE checklist for cross-sectional studies.

	Item No	Recommendation	Page No
**Title and abstract**	1	(*a*) Indicate the study's design with a commonly used term in the title or the abstract	1
		(*b*) Provide in the abstract an informative and balanced summary of what was done and what was found	Abstract (separate page)
**Introduction**			
Background/rationale	2	Explain the scientific background and rationale for the investigation being reported	3
Objectives	3	State specific objectives, including any prespecified hypotheses	3,4
**Methods**			
Study design	4	Present key elements of study design early in the paper	4,5
Setting	5	Describe the setting, locations, and relevant dates, including periods of recruitment, exposure, follow-up, and data collection	4,5
Participants	6	(*a*) Give the eligibility criteria, and the sources and methods of selection of participants	4,5,6, SDC
Variables	7	Clearly define all outcomes, exposures, predictors, potential confounders, and effect modifiers. Give diagnostic criteria, if applicable	5,6, SDC
Data sources/ measurement	8	For each variable of interest, give sources of data and details of methods of assessment (measurement). Describe comparability of assessment methods if there is more than one group	4,5,6, SDC
Bias	9	Describe any efforts to address potential sources of bias	SDC
Study size	10	Explain how the study size was arrived at	5
Quantitative variables	11	Explain how quantitative variables were handled in the analyses. If applicable, describe which groupings were chosen and why	SDC
Statistical methods	12	(*a*) Describe all statistical methods, including those used to control for confounding	SDC
		(*b*) Describe any methods used to examine subgroups and interactions	6, SDC
		(*c*) Explain how missing data were addressed	SDC
		(*d*) If applicable, describe analytical methods taking account of sampling strategy	5

		(*e*) Describe any sensitivity analyses	6
**Results**			
Participants	13	(a) Report numbers of individuals at each stage of study—eg numbers potentially eligible, examined for eligibility, confirmed eligible, included in the study, completing follow-up, and analysed	SDC
		(b) Give reasons for non-participation at each stage	SDC
		(c) Consider use of a flow diagram	SDC
Descriptive data	14	(a) Give characteristics of study participants (eg demographic, clinical, social) and information on exposures and potential confounders	Tables
		(b) Indicate number of participants with missing data for each variable of interest	SDC
Outcome data	15	Report numbers of outcome events or summary measures	Tables
Main results	16	(*a*) Give unadjusted estimates and, if applicable, confounder-adjusted estimates and their precision (eg, 95% confidence interval). Make clear which confounders were adjusted for and why they were included	Tables
		(*b*) Report category boundaries when continuous variables were categorized	Tables
		(*c*) If relevant, consider translating estimates of relative risk into absolute risk for a meaningful time period	Not applicable
Other analyses	17	Report other analyses done—eg analyses of subgroups and interactions, and sensitivity analyses	Tables
**Discussion**			
Key results	18	Summarise key results with reference to study objectives	12, 13
Limitations	19	Discuss limitations of the study, taking into account sources of potential bias or imprecision. Discuss both direction and magnitude of any potential bias	14
Interpretation	20	Give a cautious overall interpretation of results considering objectives, limitations, multiplicity of analyses, results from similar studies, and other relevant evidence	12, 13, 15
Generalisability	21	Discuss the generalisability (external validity) of the study results	13, 14
**Other information**			

Funding	22	Give the source of funding and the role of the funders for the present study and, if applicable, for the original study on which the present article is based	16

Supplemental Digital Content 2. Cohort diagram.

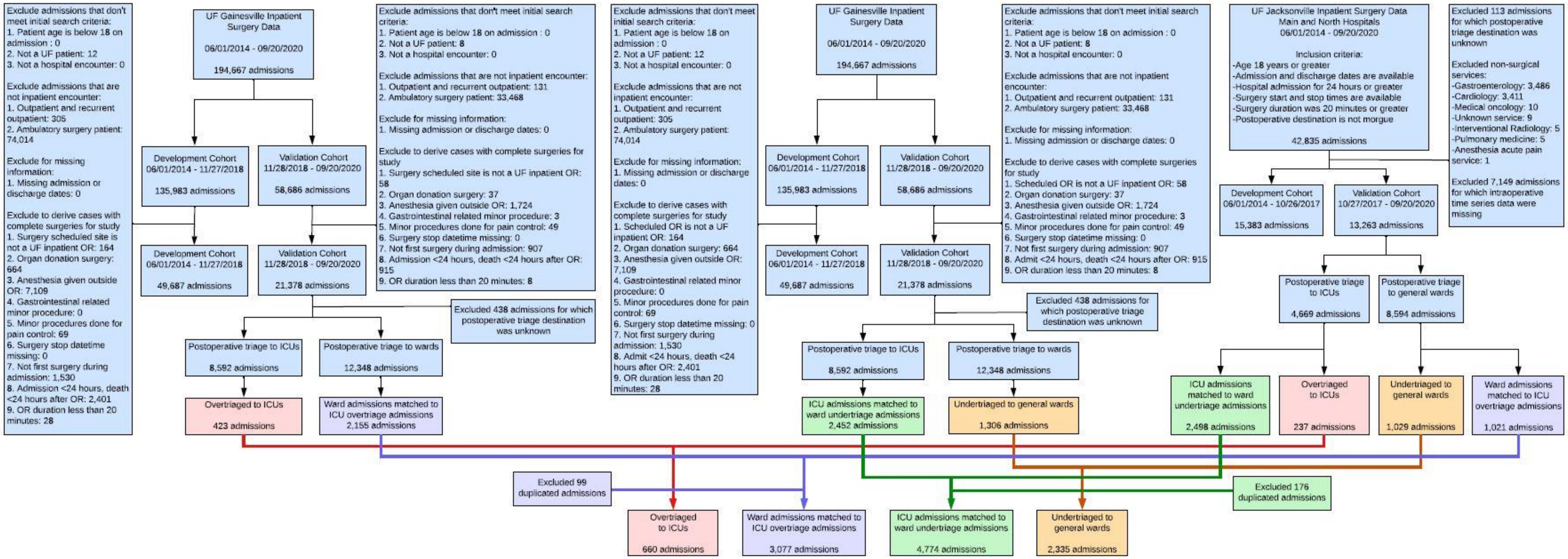

Supplemental Digital Content 3. Supplemental methods.

Clustering Methods

The clustering analyses were intended to understand sociodemographic phenotypes in previously defined cohorts without seeking to develop and validate a clustering algorithm that could predict cluster labels in new, unseen data. Therefore, all clustering analyses were performed on all relevant admissions without splitting them into training and testing cohorts. Phenotyping was performed with an open-source K-means clustering algorithm (documentation is available at: https://scikit-learn.org/stable/modules/generated/sklearn.cluster.KMeans.html, accessed 12 May 2022) using standard, default hyperparameters inherent to the algorithm. K-means was chosen as the clustering approach because it offers unique advantages for exploratory classifications of patient states.[1] Like other centroid-based clustering algorithms, K-means is sensitive to outliers; the source datasets for this study were subjected to outlier imputation, defining observations in the top and bottom 1% of each continuous variable's distribution as outliers, as previously described.[2-4] K-means algorithms minimize squared Euclidean distance between points within a cluster (within-cluster sum of squares) while maximizing the squared distance between the geometric center of each cluster (between-cluster sum of squares). Investigators choose the number of clusters, K. In this study, the optimal number of clusters was determined by calculating the variance within clusters for a range of 1-9 clusters and identifying the inflection point at which a greater number of clusters (and attendant decrease in cluster sizes) would not substantially tighten the clusters (decrease the within-cluster sum of squares), as visualized for both overtriage and undertriage clustering in **eFigure 2**. These results were consistent with clinical intuition regarding cohort size and characteristics, adhering to the notion from Preud'homme and colleagues[5] that "despite the immense progress enabled by artificial intelligence in recent years, human experience and intuition remain the best judge in cluster analysis." Once K is chosen, the algorithm randomly selects K data points as centroids. Next, the algorithm calculates similarity between each point and each centroid and groups each point with its nearest centroid. Then, centroid positions are updated by calculating the geometric mean among its data points. This

process is repeated until centroid positions and data point groupings remain constant. To visualize the overtriage and undertriage phenotypes, two-dimensional t-distributed stochastic neighbor embedding (t-SNE) was performed with an open-source algorithm (documentation is available at: https://scikit-learn.org/stable/modules/generated/sklearn.manifold.TSNE.html, accessed 12 May 2022). Briefly, t-SNE is a method for understanding patterns in complex, high-dimensional data (in this study, high dimensionality was conferred by having many variables represent few postoperative admissions) by calculating pair-wise probability distributions in which similar data points are assigned higher probabilities while dissimilar data points are assigned lower probabilities, and then mapping the distributions into two-dimensional (as in this study) or three-dimensional space for visual interpretation.

Statistical Analyses

To determine whether previously observed associations among overtriage, undertriage, mortality, morbidity, and value of care are reproduced in the new, harmonized dataset, these associations are tested and reported, along with an expanded analysis of sociodemographic factors that includes primary payer. Both hospital mortality and discharge to hospice were considered observed mortalities in the value of care analyses. Admissions with missing data were excluded unless otherwise noted in the results, as reported by the raw number and percentage of admissions with missing or unknown values. Categorical variables were compared by Fisher's Exact test and reported as raw numbers with percentages. Continuous variables were compared by the Kruskal-Wallis test and reported as median values with interquartile ranges. All statistical tests were 2-sided with alpha=.05. Overtriaged admissions were compared pairwise with both overtriage controls and undertriaged admissions, undertriaged admissions were compared pairwise with both undertriage controls and overtriaged admissions, and each phenotype was compared pairwise with other phenotypes. All outcome analyses were adjusted for multiple comparisons by applying Benjamini-Hochberg procedure separately for outcomes describing the entire cohort, overtriage

phenotypes, and undertriage phenotypes. Clustering and statistical analyses were performed using Python 3.8 software.

Model Features

Feature	Type	Use in models	
		Prolonged ICU stay	Hospital mortality
**Static variables**			
ADI national rank	Continuous	✓	✓
ADI state rank	Continuous		✓
Admission as a trauma alert	Binary		✓
Age	Continuous		✓
ASA score[b]	Continuous	✓	✓
Charlson comorbidity index score	Continuous		✓
Current smoker	Binary		✓
Elective admission priority	Binary	✓	✓
Elective surgery priority	Binary		✓
Emergent admission priority	Binary		✓
Emergent surgery priority	Binary		✓
Female sex	Binary		✓
Former smoker	Binary		✓
Hospital location prior to surgery[b]	Nominal	✓	✓
Initial presentation to the emergency department	Binary		✓
Intraoperative red cell transfusion	Binary	✓	✓
Intraoperative red cell transfusion volume	Continuous		✓
Male sex	Binary		✓
Never smoker	Binary		✓
Payer			
Blue Cross Blue Shield	Binary		✓
Commercial	Binary		✓
Federal, non-Centers for Medicare & Medicaid	Binary		✓
Managed care	Binary		✓
Medicaid	Binary		✓
Medicaid Health Maintenance Organization	Binary		✓
Medicare	Binary		✓
Medicare Health Maintenance Organization	Binary		✓
Preoperative red cell transfusion	Binary		✓
Preoperative red cell transfusion volume	Continuous		✓

Present on admission			
Acquired immune deficiency syndrome	Binary		✓
Diabetes with complications	Binary		✓
Diabetes without complications	Binary		✓
Peptic ulcer disease	Binary		✓
Cancer	Binary		✓
Cerebrovascular disease	Binary		✓
Congestive heart failure	Binary		✓
Dementia	Binary		✓
History of myocardial infarction	Binary		✓
Metastatic cancer	Binary		✓
Moderate or severe liver disease	Binary		✓
Paraplegia or hemiplegia	Binary		✓
Primary service[b]	Nominal	✓	✓
Primary surgeon	Nominal	✓	✓
Primary surgery CPT code	Nominal	✓	✓
Smoking status unknown	Binary		✓
SOFA score immediately prior to surgery[b]	Continuous	✓	✓
SOFA score on admission[b]	Continuous	✓	✓
Urgent admission priority	Binary		✓
Urgent surgery priority	Binary		✓
**Intraoperative time series variables**			
Diastolic blood pressure, mmHg	Continuous	✓	✓
End-tidal CO_2 ($ETCO_2$)	Continuous	✓	✓
Heart rate, bpm	Continuous	✓	✓
Peak Inspiratory Pressure (PIP)	Continuous	✓	✓
Peripheral capillary oxygen saturation (SPO_2)	Continuous	✓	✓
Positive End-expiratory Pressure (PEEP)	Continuous	✓	✓
Respiratory Rate	Continuous	✓	✓
Systolic blood pressure, mmHg	Continuous	✓	✓
Tidal volume, mL	Continuous	✓	✓
Temperature (^oC)	Continuous	✓	✓

[b]For model training and testing, this variable was also broken down into binary variables for each level or category (e.g., Orthopedic Surgery service: yes/no, Thoracic and Cardiovascular Surgery service: yes/no; SOFA score on admission 2 or greater: yes/no, SOFA score on admission 3 or greater: yes/no.

ADI: area deprivation index, ASA: American Society of Anaesthesiologists, CPT: Current Procedural Terminology, SOFA: sequential organ failure assessment.

References

1. Komorowski M, Celi LA, Badawi O, Gordon AC, Faisal AA. The Artificial Intelligence Clinician learns optimal treatment strategies for sepsis in intensive care. *Nat Med.* 2018;24(11):1716-1720.
2. Nowak-Brzezinska A, Lazarz W. Qualitative Data Clustering to Detect Outliers. *Entropy (Basel).* 2021;23(7).
3. Loftus TJ, Ruppert MM, Ozrazgat-Baslanti T, et al. Association of Postoperative Undertriage to Hospital Wards With Mortality and Morbidity. *JAMA Netw Open.* 2021;4(11):e2131669.
4. Loftus TJ, Balch JA, Ruppert MM, et al. Aligning Patient Acuity with Resource Intensity after Major Surgery: A Scoping Review. *Ann Surg.* 2021.
5. Preud'homme G, Duarte K, Dalleau K, et al. Head-to-head comparison of clustering methods for heterogeneous data: a simulation-driven benchmark. *Sci Rep.* 2021;11(1):4202.

Supplemental Digital Content 4. Illness severity for postoperative admissions that were overtriaged to intensive care units, undertriaged to hospital wards, and-risk matched controls.

**Illness severity indicators**	**Overtriage** N=660 No. (%)	**Overtriage controls** N=3,077 No. (%)	**Undertriage** N=2,335 No. (%)	**Undertriage controls** N=4,774 No. (%)	**P[a]**	**P[b]**	**P[c]**
Admission SOFA score, median [IQR]	0.0 [0.0-2.0]	0.0 [0.0-0.0]	0.0 [0.0-3.0]	1.0 [0.0-3.0]	**<.001**	**<.001**	.23
Admission SOFA score 2 or greater	227 (34.4)	423 (13.7)	801 (34.3)	2279 (47.7)	**<.001**	**<.001**	.96
Preoperative SOFA score, median [IQR]	0.0 [0.0-1.0]	0.0 [0.0-0.0]	0.0 [0.0-1.0]	0.0 [0.0-2.0]	**<.001**	**<.001**	.43
Preoperative SOFA score 2 or greater	113 (17.1)	60 (1.9)	429 (18.4)	1337 (28.0)	**<.001**	**<.001**	.49
ASA score, median [IQR]	3.0 [2.0-3.0]	3.0 [2.0-3.0]	3.0 [3.0-3.0]	3.0 [3.0-3.0]	**<.001**	**<.001**	**<.001**
ASA score 3 or greater	368 (55.8)	2052 (66.7)	2164 (92.7)	4130 (86.5)	**<.001**	**<.001**	**<.001**
Charlson comorbidity index score, median [IQR]	0.0 [0.0-2.0]	0.0 [0.0-2.0]	2.0 [1.0-4.0]	1.0 [0.0-3.0]	.77	**<.001**	**<.001**
Admission priority							
Elective	410 (62.1)	1739 (56.5)	637 (27.3)	2089 (43.8)	**.008**	**<.001**	**<.001**
Urgent	6 (0.9)	106 (3.4)	117 (5.0)	140 (2.9)	**<.001**	**<.001**	**<.001**
Emergent	243 (36.8)	1232 (40.0)	1581 (67.7)	2545 (53.3)	.14	**<.001**	**<.001**
Unknown	1 (0.2)	0 (0.0)	0 (0.0)	0 (0.0)	.18	>.99	.22
Surgery priority							
Elective	498 (75.5)	2378 (77.3)	1684 (72.1)	3613 (75.7)	.31	**.001**	.09
Urgent	44 (6.7)	181 (5.9)	140 (6.0)	241 (5.0)	.47	.10	.52
Emergent	118 (17.9)	518 (16.8)	511 (21.9)	920 (19.3)	.53	**.01**	**.03**
Had preoperative red cell transfusion	1 (0.2)	23 (0.7)	142 (6.1)	127 (2.7)	.11	**<.001**	**<.001**
Had intraoperative red cell transfusion	0 (0.0)	13 (0.4)	46 (2.0)	95 (2.0)	.14	>.99	**<.001**

IQR: interquartile range, SOFA: sequential organ failure assessment, ASA: American Society of Anesthesiologists, P values correspond to significance tests comparing the appropriate triage, undertriage, and control cohorts by each variable listed in the "Illness severity indicators" column. [a]Overtriage versus overtriage controls, which were ward admissions with risk profiles similar to those of overtriaged intensive care unit admissions. [b]Undertriage versus undertriage controls, which were ICU admissions with risk profiles similar to those of undertriaged ward admissions. [c]Overtriage versus undertriage.

Supplemental Digital Content 5. Primary surgical services of postoperative admissions that were overtriaged to intensive care units, undertriaged to hospital wards, and-risk matched controls.

**Primary surgical service**	**Overtriage** N=660 No. (%)	**Overtriage Controls** N=3,077 No. (%)	**Undertriage** N=2,335 No. (%)	**Undertriage Controls** N=4,774 No. (%)	**P[a]**	**P[b]**	**P[c]**
Breast, Melanoma, Sarcoma, Endocrine	11 (1.7)	41 (1.3)	21 (0.9)	9 (0.2)	.47	**<.001**	.13
Burn Surgery	24 (3.6)	15 (0.5)	10 (0.4)	75 (1.6)	**<.001**	**<.001**	**<.001**
Colorectal Surgery	17 (2.6)	100 (3.2)	36 (1.5)	34 (0.7)	.46	**.001**	.09
General Surgery	36 (5.5)	151 (4.9)	187 (8.0)	367 (7.7)	.56	.64	**.03**
Gynecologic Surgery	21 (3.2)	431 (14.0)	88 (3.8)	31 (0.6)	**<.001**	**<.001**	.56
Minimally Invasive Surgery	7 (1.1)	117 (3.8)	21 (0.9)	15 (0.3)	**<.001**	**.002**	.65
Neurosurgery	94 (14.2)	291 (9.5)	241 (10.3)	1074 (22.5)	**<.001**	**<.001**	**.006**
Ophthalmology	5 (0.8)	13 (0.4)	30 (1.3)	17 (0.4)	.35	**<.001**	.31
Oral Surgery	19 (2.9)	128 (4.2)	106 (4.5)	133 (2.8)	.15	**<.001**	.06
Orthopedic Surgery	137 (20.8)	923 (30.0)	475 (20.3)	583 (12.2)	**<.001**	**<.001**	.83
Otolaryngology	56 (8.5)	73 (2.4)	56 (2.4)	253 (5.3)	**<.001**	**<.001**	**<.001**
Pancreas and Biliary Surgery	14 (2.1)	11 (0.4)	19 (0.8)	77 (1.6)	**<.001**	**.006**	**.009**
Pediatric Surgery (age 18 years or greater)	1 (0.2)	16 (0.5)	14 (0.6)	4 (0.1)	.34	**<.001**	.22
Podiatry	4 (0.6)	56 (1.8)	77 (3.3)	94 (2.0)	**.03**	**<.001**	**<.001**
Plastic and Reconstructive Surgery	17 (2.6)	58 (1.9)	54 (2.3)	61 (1.3)	.28	**.002**	.67
Thoracic and Cardiovascular Surgery	5 (0.8)	3 (0.1)	51 (2.2)	603 (12.6)	**.006**	**<.001**	**.01**
Transplant Surgery	4 (0.6)	45 (1.5)	45 (1.9)	27 (0.6)	.09	**<.001**	**.02**
Trauma and Acute Care Surgery	66 (10.0)	213 (6.9)	255 (10.9)	530 (11.1)	**.009**	.84	.52
Urology	65 (9.8)	289 (9.4)	181 (7.8)	148 (3.1)	.71	**<.001**	.09
Vascular Surgery	57 (8.6)	103 (3.3)	368 (15.8)	639 (13.4)	**<.001**	**.007**	**<.001**

P values correspond to significance tests comparing cohorts by each variable in the "Primary surgical service" column. [a]Overtriage versus overtriage controls, which were ward admissions with risk profiles similar to those of overtriaged intensive care unit admissions. [b]Undertriage versus undertriage controls, which were ICU admissions with risk profiles similar to those of undertriaged ward admissions. [c]Overtriage versus undertriage.

Supplemental Digital Content 6. Elbow plot illustrating associations between number of clusters and the sum of squared differences. **A:** Elbow plot for clustering within the overtriage cohort. **B:** Elbow plot for clustering within the undertriage cohort.

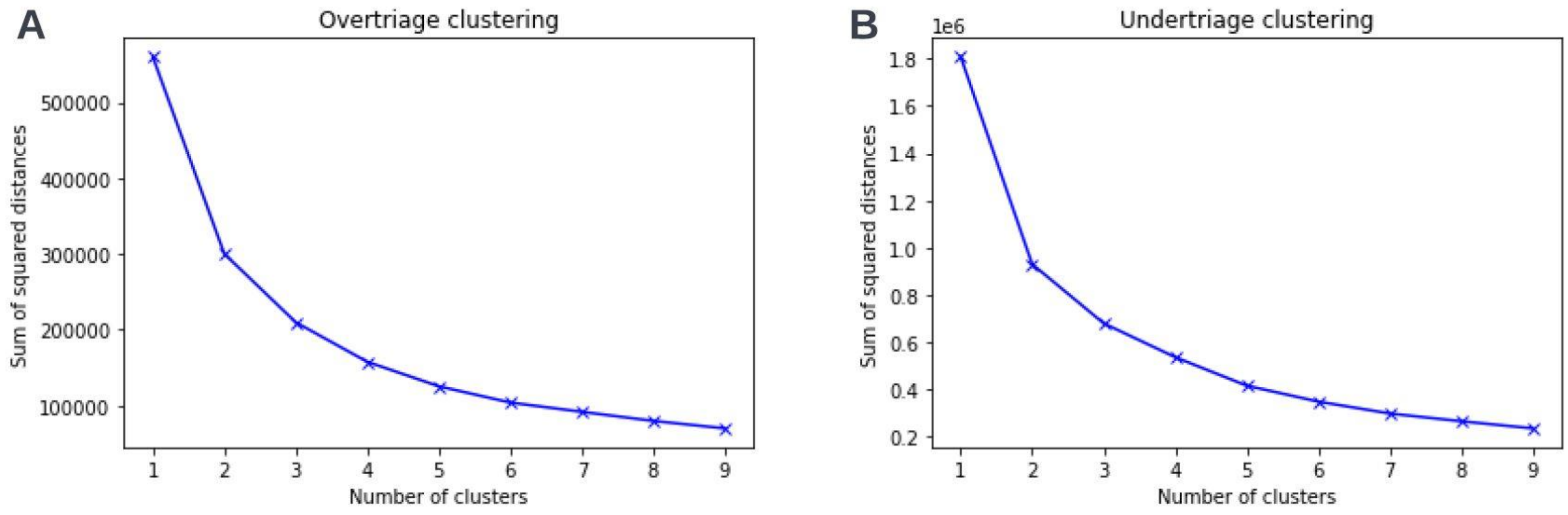

Supplemental Digital Content 7. T-distributed stochastic neighbor embedding plots of overtriage and undertriage phenotypes. **A:** Cluster phenotypes within the overtriage cohort. **B:** Cluster phenotypes within the undertriage cohort.

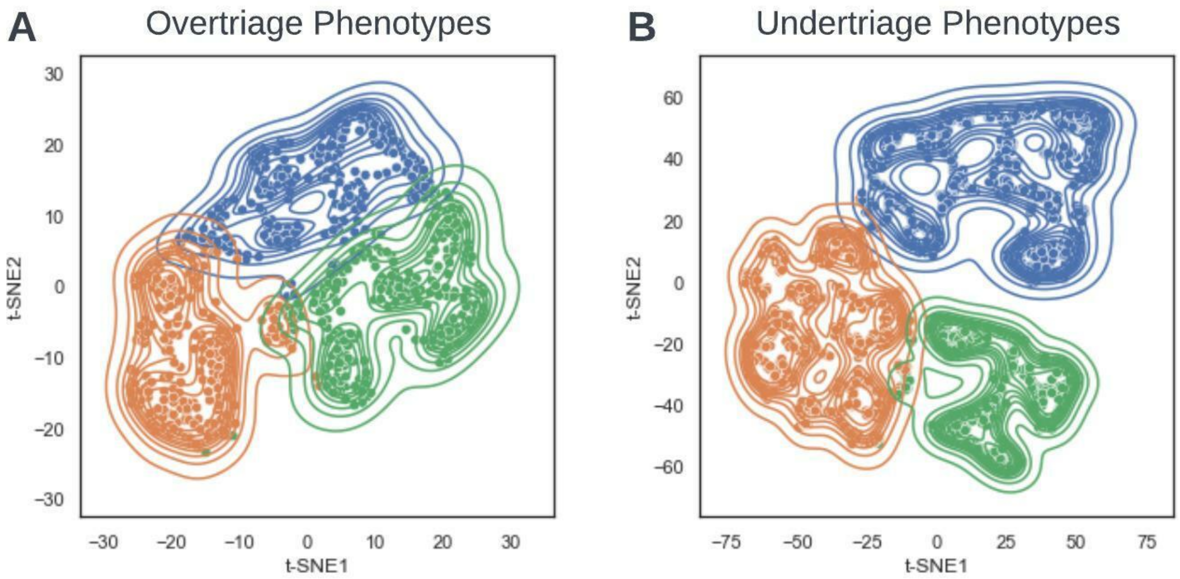

Supplemental Digital Content 8. Illness severity indicators of overtriage phenotypes.

**Illness severity indicators**	**Alpha phenotype** N=233 No. (%)	**Beta phenotype** N=218 No. (%)	**Gamma phenotype** N=209 No. (%)	**P[a]**	**P[b]**	**P[c]**
Admission SOFA score, median [IQR]	0.0 [0.0-2.0]	1.0 [0.0-3.0]	0.0 [0.0-2.0]	**.009**	.23	.17
Admission SOFA score 2 or greater	66 (28.3)	90 (41.3)	71 (34.0)	**.004**	.13	.22
Preoperative SOFA score, median [IQR]	0.0 [0.0-1.0]	0.0 [0.0-1.0]	0.0 [0.0-1.0]	.76	.66	.46
Preoperative SOFA score 2 or greater	36 (15.5)	41 (18.8)	36 (17.2)	.38	.71	.70
ASA score, median [IQR]	3.0 [3.0-3.0]	3.0 [2.0-3.0]	2.0 [2.0-3.0]	**<.001**	**<.001**	**<.001**
ASA score 3 or greater	175 (75.1)	120 (55.0)	73 (34.9)	**<.001**	**<.001**	**<.001**
Charlson comorbidity index score, median [IQR]	1.0 [0.0-3.0]	0.0 [0.0-2.0]	0.0 [0.0-1.0]	**<.001**	**<.001**	**<.001**
Admission priority						
Elective	174 (74.7)	158 (72.5)	78 (37.3)	.67	**<.001**	**<.001**
Urgent	2 (0.9)	0 (0.0)	4 (1.9)	.50	.06	.43
Emergent	57 (24.5)	59 (27.1)	127 (60.8)	.59	**<.001**	**<.001**
Unknown	0 (0.0)	1 (0.5)	0 (0.0)	.48	>.99	>.99
Surgery priority						
Elective	195 (83.7)	178 (81.7)	125 (59.8)	.62	**<.001**	**<.001**
Urgent	10 (4.3)	9 (4.1)	25 (12.0)	>.99	**.004**	**.004**
Emergent	28 (12.0)	31 (14.2)	59 (28.2)	.58	**<.001**	**<.001**
Had preoperative red cell transfusion	0 (0.0)	0 (0.0)	1 (0.5)	>.99	.49	.47
Had intraoperative red cell transfusion	0 (0.0)	0 (0.0)	0 (0.0)	>.99	>.99	>.99

IQR: interquartile range, SOFA: sequential organ failure assessment, ASA: American Society of Anesthesiologists, P values correspond to significance tests comparing the appropriate triage, undertriage, and control cohorts by each variable listed in the "Illness severity indicators" column. [a]Alpha phenotype versus beta phenotype. [b]Alpha phenotype versus gamma phenotype. [c]Beta phenotype versus gamma phenotype.

Supplemental Digital Content 9. Primary surgical services of overtriage phenotypes.

**Primary surgical service**	**Alpha phenotype** N=233 No. (%)	**Beta phenotype** N=218 No. (%)	**Gamma phenotype** N=209 No. (%)	**P[a]**	**P[b]**	**P[c]**
Breast, Melanoma, Sarcoma, Endocrine	7 (3.0)	3 (1.4)	1 (0.5)	.34	.07	.62
Burn Surgery	6 (2.6)	8 (3.7)	10 (4.8)	.59	.31	.64
Colorectal Surgery	9 (3.9)	6 (2.8)	2 (1.0)	.60	.07	.29
General Surgery	14 (6.0)	13 (6.0)	9 (4.3)	>.99	.52	.51
Gynecologic Surgery	8 (3.4)	8 (3.7)	5 (2.4)	>.99	.58	.58
Minimally Invasive Surgery	3 (1.3)	2 (0.9)	2 (1.0)	>.99	>.99	>.99
Neurosurgery	24 (10.3)	48 (22.0)	22 (10.5)	**<.001**	>.99	**.002**
Ophthalmology	2 (0.9)	0 (0.0)	3 (1.4)	.50	.67	.12
Oral Surgery	1 (0.4)	7 (3.2)	11 (5.3)	**.03**	**.002**	.34
Orthopedic Surgery	43 (18.5)	41 (18.8)	53 (25.4)	>.99	.08	.13
Otolaryngology	20 (8.6)	18 (8.3)	18 (8.6)	>.99	>.99	>.99
Pancreas and Biliary Surgery	9 (3.9)	2 (0.9)	3 (1.4)	.06	.15	.68
Pediatric Surgery (age 18 years or greater)	0 (0.0)	0 (0.0)	1 (0.5)	>.99	.47	.49
Podiatry	0 (0.0)	1 (0.5)	3 (1.4)	.48	.11	.36
Plastic and Reconstructive Surgery	4 (1.7)	6 (2.8)	7 (3.3)	.53	.36	.78
Thoracic and Cardiovascular Surgery	2 (0.9)	2 (0.9)	1 (0.5)	>.99	>.99	>.99
Transplant Surgery	1 (0.4)	2 (0.9)	1 (0.5)	.61	>.99	>.99
Trauma and Acute Care Surgery	18 (7.7)	16 (7.3)	32 (15.3)	>.99	**.02**	**.01**
Urology	30 (12.9)	20 (9.2)	15 (7.2)	.23	.06	.49
Vascular Surgery	32 (13.7)	15 (6.9)	10 (4.8)	**.02**	**.002**	.41

P values correspond to significance tests comparing cohorts by each variable in the “Primary surgical service” column. [a]Alpha phenotype versus beta phenotype. [b]Alpha phenotype versus gamma phenotype. [c]Beta phenotype versus gamma phenotype.

Supplemental Digital Content 10. Outcomes of overtriage phenotypes.

**Outcomes**	**Alpha phenotype** N=233 No. (%)	**Beta phenotype** N=218 No. (%)	**Gamma phenotype** N=209 No. (%)	**P[a]**	**P[b]**	**P[c]**
Second surgery during admission	16 (6.9)	16 (7.3)	24 (11.5)	>.99	.45	.62
Hours between surgeries, median [IQR]	69 [44-128]	59 [44-93]	64 [42-101]	.95	>.99	>.99
Emergent second surgery	2 (0.9)	2 (0.9)	4 (1.9)	>.99	.82	.82
Had postoperative red cell transfusion	12 (5.2)	12 (5.5)	6 (2.9)	>.99	.80	.68
Red cell transfusion during admission	12 (5.2)	12 (5.5)	6 (2.9)	>.99	.80	.68
AKI with rapid reversal	18 (7.7)	18 (8.3)	9 (4.3)	>.99	.59	.47
Persistent AKI with renal recovery	4 (1.7)	0 (0.0)	1 (0.5)	.48	.80	.84
Persistent AKI without renal recovery	13 (5.6)	8 (3.7)	6 (2.9)	.80	.68	>.99
ICU admission for ≥48 hours	57 (24.5)	50 (22.9)	41 (19.6)	>.99	.68	.82
Mechanical ventilation for ≥48 hours	2 (0.9)	1 (0.5)	0 (0.0)	>.99	.84	>.99
ICU length of stay (days), median [IQR]	1.2 [0.8-2.0]	1.0 [0.7-2.0]	0.9 [0.0-1.8]	.45	**<.001**	.08
Hospital length of stay (days), median [IQR]	1.9 [1.1-3.2]	1.7 [7.9-2.9]	1.9 [1.2-3.4]	.18	>.99	.10
Hospital mortality	1 (0.4)	1 (0.5)	0 (0.0)	>.99	>.99	>.99
Discharge to hospice	0 (0.0)	0 (0.0)	0 (0.0)	>.99	>.99	>.99
Professional service charges, $K, median [IQR]	13.9 [10.6-20.1]	14.8 [10.3-21.7]	11.3 [8.2-16.8]	.84	**.004**	**.002**
Hospital admission charges, $K, median [IQR]	69.5 [50.2-101.5]	74.9 [50.5-100.8]	67.8 [49.5-105.2]	>.99	>.99	.80
Hospital admission costs, $K, median [IQR]	17.1 [11.8-25.8]	16.3 [12.1-22.4]	15.2 [10.5-21.5]	.80	.10	.45
Value of care, median [IQR]	0.5 [0.3-0.7]	0.5 [0.4-0.7]	0.5 [0.4-0.8]	.80	.10	.45

IQR: interquartile range, ICU: intensive care unit, AKI: acute kidney injury. P values were adjusted for multiple comparisons using the Benjamini-Hochberg procedure. P values correspond to significance tests comparing cohorts by each variable in the “Outcomes” column. [a]Alpha phenotype versus beta phenotype. [b]Alpha phenotype versus gamma phenotype. [c]Beta phenotype versus gamma phenotype.

Supplemental Digital Content 11. Illness severity indicators of undertriage phenotypes.

**Illness severity indicators**	**Delta phenotype** N=992 No. (%)	**Epsilon phenotype** N=820 No. (%)	**Zeta phenotype** N=523 No. (%)	**P[a]**	**P[b]**	**P[c]**
Admission SOFA score, median [IQR]	0.0 [0.0-2.0]	0.0 [0.0-3.0]	0.0 [0.0-3.0]	.23	**<.001**	**.01**
Admission SOFA score 2 or greater	312 (31.5)	277 (33.8)	212 (40.5)	.31	**<.001**	**.01**
Preoperative SOFA score, median [IQR]	0.0 [0.0-0.0]	0.0 [0.0-1.0]	0.0 [0.0-1.0]	.44	**.006**	**.04**
Preoperative SOFA score 2 or greater	174 (17.5)	139 (17.0)	116 (22.2)	.76	**.03**	**.02**
ASA score, median [IQR]	3.0 [3.0-3.0]	3.0 [3.0-3.0]	3.0 [3.0-3.0]	**<.001**	**<.001**	**.002**
ASA score 3 or greater	949 (95.7)	765 (93.3)	450 (86.0)	**.03**	**<.001**	**<.001**
Charlson comorbidity index score, median [IQR]	3.0 [1.0-5.0]	2.0 [1.0-4.0]	2.0 [0.0-4.0]	**<.001**	**<.001**	**.001**
Admission priority						
Elective	240 (24.2)	259 (31.6)	138 (26.4)	**<.001**	.35	**.04**
Urgent	40 (4.0)	51 (6.2)	26 (5.0)	**.04**	.43	.40
Emergent	712 (71.8)	510 (62.2)	359 (68.6)	**<.001**	.21	**.02**
Unknown	0 (0.0)	0 (0.0)	0 (0.0)	>.99	>.99	>.99
Surgery priority						
Elective	733 (73.9)	604 (73.7)	347 (66.3)	.92	**.002**	**.005**
Urgent	62 (6.2)	46 (5.6)	32 (6.1)	.62	>.99	.72
Emergent	197 (19.9)	170 (20.7)	144 (27.5)	.68	**<.001**	**.004**
Had preoperative red cell transfusion	60 (6.0)	44 (5.4)	38 (7.3)	.55	.38	.16
Had intraoperative red cell transfusion	22 (2.2)	12 (1.5)	12 (2.3)	.30	>.99	.29

IQR: interquartile range, SOFA: sequential organ failure assessment, ASA: American Society of Anesthesiologists, P values correspond to significance tests comparing the appropriate triage, undertriage, and control cohorts by each variable listed in the "Illness severity indicators" column. [a]Delta phenotype versus epsilon phenotype. [b]Delta phenotype versus zeta phenotype. [c]Epsilon phenotype versus zeta phenotype.

Supplemental Digital Content 12. Primary surgical services of undertriage phenotypes.

**Primary surgical service**	**Delta phenotype** N=992 No. (%)	**Epsilon phenotype** N=820 No. (%)	**Zeta phenotype** N=523 No. (%)	**P[a]**	**P[b]**	**P[c]**
Breast, Melanoma, Sarcoma, Endocrine	7 (0.7)	8 (1.0)	6 (1.1)	.61	.39	.79
Burn Surgery	4 (0.4)	2 (0.2)	4 (0.8)	.70	.46	.22
Colorectal Surgery	8 (0.8)	20 (2.4)	8 (1.5)	**.007**	.20	.33
General Surgery	45 (4.5)	94 (11.5)	48 (9.2)	**<.001**	**<.001**	.20
Gynecologic Surgery	25 (2.5)	26 (3.2)	37 (7.1)	.48	**<.001**	**.001**
Minimally Invasive Surgery	4 (0.4)	12 (1.5)	5 (1.0)	**.02**	.29	.47
Neurosurgery	91 (9.2)	102 (12.4)	48 (9.2)	**.03**	>.99	.08
Ophthalmology	11 (1.1)	11 (1.3)	8 (1.5)	.67	.48	.82
Oral Surgery	39 (3.9)	44 (5.4)	23 (4.4)	.18	.68	.44
Orthopedic Surgery	255 (25.7)	167 (20.4)	53 (10.1)	**.007**	**<.001**	**<.001**
Otolaryngology	24 (2.4)	19 (2.3)	13 (2.5)	>.99	>.99	.86
Pancreas and Biliary Surgery	7 (0.7)	9 (1.1)	3 (0.6)	.45	>.99	.39
Pediatric Surgery (age 18 years or greater)	0 (0.0)	4 (0.5)	10 (1.9)	**.04**	**<.001**	**.02**
Podiatry	27 (2.7)	22 (2.7)	28 (5.4)	>.99	**.01**	**.02**
Plastic and Reconstructive Surgery	16 (1.6)	19 (2.3)	19 (3.6)	.31	**.02**	.18
Thoracic and Cardiovascular Surgery	25 (2.5)	16 (2.0)	10 (1.9)	.43	.59	>.99
Transplant Surgery	14 (1.4)	19 (2.3)	12 (2.3)	.16	.22	>.99
Trauma and Acute Care Surgery	116 (11.7)	70 (8.5)	69 (13.2)	**.03**	.41	**.008**
Urology	90 (9.1)	53 (6.5)	38 (7.3)	**.04**	.25	.58
Vascular Surgery	184 (18.5)	103 (12.6)	81 (15.5)	**<.001**	.16	.14

P values correspond to significance tests comparing cohorts by each variable in the "Primary surgical service" column. [a]Delta phenotype versus epsilon phenotype. [b]Delta phenotype versus zeta phenotype. [c]Epsilon phenotype versus zeta phenotype.

Supplemental Digital Content 13. Outcomes of undertriage phenotypes.

**Outcomes**	**Delta phenotype** N=992 No. (%)	**Epsilon phenotype** N=820 No. (%)	**Zeta phenotype** N=523 No. (%)	**P[a]**	**P[b]**	**P[c]**
Second surgery during admission	188 (19.0)	181 (22.1)	106 (20.3)	.43	.93	.93
Hours between surgeries, median [IQR]	89.5 [48.0-145.8]	87.0 [48.0-141.0]	92.0 [49.0-148.5]	.93	.96	.96
Emergent second surgery	51 (5.1)	44 (5.4)	22 (4.2)	.96	.93	.93
Had postoperative red cell transfusion	168 (16.9)	140 (17.1)	84 (16.1)	.99	.94	.93
Red cell transfusion during admission	220 (22.2)	175 (21.3)	112 (21.4)	.93	.96	>.99
AKI with rapid reversal	126 (12.7)	104 (12.7)	45 (8.6)	>.99	.23	.23
Persistent AKI with renal recovery	76 (7.7)	47 (5.7)	36 (6.9)	.43	.93	.93
Persistent AKI without renal recovery	65 (6.6)	49 (6.0)	30 (5.7)	.93	.93	.96
ICU admission for ≥48 hours	99 (10.0)	84 (10.2)	39 (7.5)	.96	.43	.43
Mechanical ventilation for ≥48 hours	20 (2.0)	15 (1.8)	16 (3.1)	.96	.67	.59
ICU length of stay (days), median [IQR]	0.0 [0.0-0.0]	0.0 [0.0-0.0]	0.0 [0.0-0.0]	.93	.18	.23
Hospital length of stay (days), median [IQR]	4.5 [2.9-7.8]	4.0 [2.4-6.9]	4.2 [2.2-8.0]	**.007**	.43	.80
Hospital mortality	19 (1.9)	12 (1.5)	5 (1.0)	.93	.59	.93
Discharge to hospice	23 (2.3)	22 (2.7)	6 (1.1)	.93	.59	.43
Professional service charges, $K, median [IQR]	16.0 [10.6-24.9]	16.1 [10.1-27.3]	14.8 [9.4-25.2]	.96	.43	.43
Hospital admission charges, $K, median [IQR]	105.7 [72.6-158.1]	103.8 [71.8-167.7]	105.7 [67.6-164.1]	.96	.93	.93
Hospital admission costs, $K, median [IQR]	25.6 [17.2-39.1]	24.0 [15.8-39.9]	23.9 [13.9-39.4]	.59	.43	.93
Value of care, median [IQR]	0.7 [0.5-1.1]	0.8 [0.5-1.2]	0.8 [0.5-1.4]	.93	.93	.96

IQR: interquartile range, ICU: intensive care unit, AKI: acute kidney injury. P values were adjusted for multiple comparisons using the Benjamini-Hochberg procedure. P values correspond to significance tests comparing cohorts by each variable in the "Outcomes" column. [a]Delta phenotype versus epsilon phenotype. [b]Delta phenotype versus zeta phenotype. [c]Epsilon phenotype versus zeta phenotypes.
